# Glioma-on-a-Chip Models

**DOI:** 10.3390/mi12050490

**Published:** 2021-04-26

**Authors:** Merve Ustun, Sajjad Rahmani Dabbagh, Irem Sultan Ilci, Tugba Bagci-Onder, Savas Tasoglu

**Affiliations:** 1 Graduate School of Sciences and Engineering, Koc University, Sariyer, 34450 Istanbul, Turkey; mustun20@ku.edu.tr; 2 Department of Mechanical Engineering, Koç University, Sariyer, 34450 Istanbul, Turkey; sdabbagh19@ku.edu.tr; 3 Koç University Arçelik Research Center for Creative Industries (KUAR), Koç University, Sariyer, 34450 Istanbul, Turkey; 4 Department of Bioengineering, Yildiz Technical University, 34220 Istanbul, Turkey; iremilci@gmail.com; 5 Brain Cancer Research and Therapy Lab, Koç University School of Medicine, 34450 Istanbul, Turkey; tuonder@ku.edu.tr; 6 Koç University Research Center for Translational Medicine, Koç University, Sariyer, 34450 Istanbul, Turkey; 7 Center for Life Sciences and Technologies, Bogazici University, Bebek, 34342 Istanbul, Turkey; 8 Boğaziçi Institute of Biomedical Engineering, Boğaziçi University, Çengelköy, 34684 Istanbul, Turkey

**Keywords:** organ-on-a-chip, glioma, brain, tissue-on-a-chip, disease-on-a-chip, tumor-on-a-chip

## Abstract

Glioma, as an aggressive type of cancer, accounts for virtually 80% of malignant brain tumors. Despite advances in therapeutic approaches, the long-term survival of glioma patients is poor (it is usually fatal within 12–14 months). Glioma-on-chip platforms, with continuous perfusion, mimic in vivo metabolic functions of cancer cells for analytical purposes. This offers an unprecedented opportunity for understanding the underlying reasons that arise glioma, determining the most effective radiotherapy approach, testing different drug combinations, and screening conceivable side effects of drugs on other organs. Glioma-on-chip technologies can ultimately enhance the efficacy of treatments, promote the survival rate of patients, and pave a path for personalized medicine. In this perspective paper, we briefly review the latest developments of glioma-on-chip technologies, such as therapy applications, drug screening, and cell behavior studies, and discuss the current challenges as well as future research directions in this field.

## 1. Introduction

Being responsible for almost 3% of cancer-related death annually in the U.S. [1], glioma is the most aggressive form of brain tumor [2]. Since nearly 49.4% of glioma patients are in the range of 20 to 64 years old [1], i.e., the economically productive portion of the population, so glioma has a considerable socioeconomic impact, highlighting the importance of early diagnosis and effective treatment. From a pathology viewpoint, possession of ultrastructural, immunohistochemical, and histological evidence of glial differentiation are the key parameters defining glioma tumors. It is widely held that glioma arises from neuroglial progenitor cells [3]. These tumors are graded from I (benign) to IV (most malignant) based on the presence of astrocytic, oligodendroglial, or ependymal cell features (the grading scheme was updated in 2016 by the World Health Organization (WHO) [3]). Common gliomas include oligodendrogliomas, infiltrative astrocytomas (glioblastoma (GBM) (grade IV), anaplastic astrocytoma (grade III), and diffuse astrocytoma (grade II)), and less frequent types such as ependymomas, pleomorphic xanthoastrocytomas, and pilocytic astrocytomas [4]. The underlying biological causes of glioma are not fully known [3]. Being exposed to ionizing radiation is the most possible risk factor correlated with GBM, whereas no evidence is reported linking mobile-phone usage to glioma. GBM can be inversely associated with allergies, atopy, and immune-related conditions; while genetic syndromes (e.g., Lynch and Li-Fraumeni syndrome) are rarely related to GBM (less than 1% of cases) [3].

The molecular mechanisms for the malignant characteristics of glioma remain elusive, however, the highly motile nature of tumor cells and their ability to disperse within the brain parenchyma are major contributing factors for poor prognosis. Given that there are still unknown mechanisms behind these very aggressive tumors, finding the most effective therapy (e.g., surgery, radiography, or dosage/combination of drugs) is still challenging. In-vitro cell cultures and in vivo animal models have been in use for decades as common laboratory methods to study gliomas. Murine models offer a platform for the investigation of the biological mechanisms of tumorigenesis as well as large-scale genomic analysis of tumor specimens for functional identification of candidate genes. However, genetic differences between mice and humans trigger inaccuracy in recapitulating human pathophysiology. For instance, roughly 50% of preclinically approved drugs turn out to be hazardous for humans, while a number of drugs that fail animal tests are nontoxic for humans, demonstrating the inadequacy of animal models and the importance of conducting studies and drug screening directly on human cells [5]. Conventional two-dimensional (2D) in vitro cell culture models provide beneficial information regarding cell analysis and drug responses, while they fail to recapitulate cell morphology, complex in-vivo structures, cell-cell, and cell-matrix interactions [6]. Since 1884 when Godlee performed the first brain tumor surgery, attempts for glioma treatment have been developed, followed by introduction of a grading scheme for glioma classification in 1926 [7]. Thenceforth, radiation therapy, surgery, and chemotherapy (mostly using temozolomide) are deemed to be standard glioma therapies [8]. Despite advances in multimodality medical therapies, virtually 70% of grade II glioma tumors still progress to grade III and IV tumors which are fatal within 12–14 months [8]. Uncertainty about the nature of glioma and the inefficiency of treatments highlights an imperative need for the development of accurate and low-cost platforms through which critical studies can be conducted. Integration of three-dimensional (3D) cell cultures with microfluidic chips recapitulates the in-vivo-like perfusion (i.e., biological fluid flow to supply nutrients and remove wastes) and structure (with porous extracellular matrix (ECM)), offers patient-driven models for personalized drug tests, and improves cell proliferation, survival, and mechano-responses, ultimately promoting our understanding of glioma and developing hybrid patient-specific drug combinations leading to superior tumor-killing capability [9].

Organ-on-chip (OOC) platforms provide a continuously perfused microenvironment, with programmable nutrient supply and waste removal, for culturing organ cells extracted from patients with high proliferation as well as viability rate, emulating in vivo-like properties of organs [10,11,12,13,14]. The word “chip” in “organ-on-chip” platforms refers to the microscale fluid handling components, including chambers, valves, membranes, and channels, used for cell culture as well as perfusion. Microfluidic chips provide laminar fluid flow on the microscale, decreasing the required amount of samples/reagents and enabling automatized control, imaging, and analysis of cultures (e.g., spatiotemporal control of molecules) [15]. Molding and 3D printing are prevalently used methods for microfluidic chip fabrication using polydimethylsiloxane (PDMS) as a biocompatible substrate for OOC applications. The versatility of microfluidic devices enables researchers to study a diverse set of biological problems including single-cell biophysical characterization, miniaturized lab-on-chip platforms, and on-chip recapitulation of the organ physiological factors [16]. Using sensors and microscopes, OOC technologies allow real-time imaging and analysis of oxygen, CO_2_, pH, metabolites (e.g., organic acids, amino acids, enzymes, and alcohol), and metabolic functions of living cells (e.g., tissue barrier integrity and cell migration) in an organ context [16,17,18]. Furthermore, theranostics, i.e., combining diagnosis and treatment, is another advantage of integrating OOC platforms with developing technologies, such as multifunctional nanoparticles for cancer treatment [16,19]. Glioma-on-chip platforms can be used to determine the most suitable radiotherapy dose, while different combinations of drugs can be tested subsequently to figure out the most effective treatment. Furthermore, integrating glioma-on-chip with other OOC platforms (e.g., lung, liver, and kidney) can shed light on the possible adverse side effects of glioma therapies on other organs [20].

The present paper reviews recent studies of glioma-on-chip for treatment and drug screening applications. Besides, the ability of OOC for cancer biology studies, such as cell invasion and cell migration, is covered. Furthermore, common microfluidic chip fabrication techniques and materials are presented.

## 2. The Biological Mechanisms of Glioblastoma

In order to develop more effective clinical therapies, a comprehensive understanding of the biological mechanisms of GBM is essential, such as processes in tumor initiation, progression, and invasion.

The genetic alterations associated with gliomas, particularly GBM, are well characterized. While some GBMs develop de novo (primary glioblastoma), some progress from lower-grade gliomas (secondary glioblastoma). In de novo GBM, common mutations are EGFR amplification or mutation, PTEN mutation, INK4A/ARF mutation, and loss of heterozygosity in 10p/10q chromosomes. On the other hand, progressive GBMs carry p53 mutation, platelet-derived growth factor receptor (PDGFR) overexpression, RB and PTEN mutations, among others [21]. With the breakthrough discovery of a mutation in a gene encoding a metabolic enzyme isocitrate dehydrogenase (IDH), GBMs can now be categorized further according to their IDH mutation status. IDH wild-type tumors are most frequently clinically defined de novo or primary GBMs, whereas IDH mutant tumors are mostly secondary GBMs. Tumors with undeterminable IDH status are classified as “not otherwise specified (NOS)” [22]. Secondary and primary GBMs have different clinical outcomes (tumors with mutated IDH2 and IDH1 have enhanced prognosis) as well as epigenetic and genetic profiles, but both tumor types display analogous histological characteristics [23]. Another key factor in stratifying GBMs is based on the gene expression of these tumors, where the GBMs have different subtypes based on their gene signatures (e.g., mesenchymal, classical, neural, and proneural) [24]. A commonly used prognostic marker in GBM therapies is the promoter hypermethylation status of a DNA repair enzyme, O6-methylguanine DNA methyltransferase (MGMT) [21]. As temozolomide (TMZ) administration and/or irradiation cause DNA damage in the proliferating tumor cells, failure to repair these damages would be advantageous for the eradication of tumor cells and a successful outcome. Indeed, tumors with hypermethylated MGMT respond to TMZ better. Therefore, in proposing effective clinical methods for GBM, all molecular features need to be considered.

Examining how tumor cells interact with the other cells in their microenvironment is also very crucial. Primary gliomas involve a diversity of malignant tumors of the central nervous system (CNS) which are thought to develop either from a subpopulation of cancer stem cells (CSCs) residing in the tissue, or from glial cells, including ependymal, microglia, oligodendrocytes, and astrocytes cells [25]. The tumor mass can be identified by extremely proliferating tumor cells, neoangiogenesis, and necrosis (surrounded by tumor cells organized in a pseudopalisading form). In GBM, tumor microenvironment contains GBM cancer stem cells (GCSCs), extracellular matrix, GBM-associated stromal cells, peripheral monocyte-derived macrophages, activated resident microglia, natural killer cells (NK cells), myeloid-derived suppressor cells (MDSCs), Treg, and immune infiltrate of T lymphocytes cells, reactive astrocytes, endothelial cells (ECs), and pericytes. The peritumoral tissue may contain tumoral cells and host peritumoral tissue cancer stem cells (PCSCs), inflammatory cells, oligodendrocytes, reactive astrocytes, extracellular matrix, GBM-associated stromal cells, ECs, and pericytes [25].

Cancer therapies have become challenging owing to the extreme heterogeneity of GBM tumors. Investigation of intra-tumor and inter-tumor heterogeneity is critical since it requires the study of a variety of biomolecular characteristics including the death and growth rate of tumor cells, the accurate identification of molecular markers, and epigenetic as well as genetic abnormalities [26,27,28]. The microenvironment of the brain includes a multitude of non-tumor cell types, such as endothelial, microglia, astrocytes, and neurons, and cells. The presence of these elements in the environment of the tumor potentially influences the progression of GBM tumors, while offering the capability to resist existing treatments (e.g., radiation) [29]. Furthermore, the development of brain tumor cells in an intricate 3D structure entangled with a brain-specific ECM and various anatomical locations of tumors further complicate the understanding and treatment of GBM [29]. For example, GBMs located in the basal ganglia and thalamus, i.e., eloquent cortices (language cortex, sensory cortex, or motor cortex) are unreachable through surgery, resulting in poor prognosis, whereas, GBMs in the temporal lobes and non-eloquent frontal are accessible by surgery with a longer survival chance of patients [30,31]. Moreover, survival rate and therapy responses partially depend on the abnormal ECM remodeling and its components, including cadherins, laminins, and integrins since it can influence drug penetration, tumor angiogenesis, endothelial and immune cells, eventually affecting tumor progression and aggressiveness [32,33]. Besides, various kinds of cellular dysfunction abet GBM cells to make themselves resistant to different anti-GBM therapies [30]. Glioma-on-chip platforms provide a promising method for shedding light on the origin of GBM, its progression process, simulating drug responses as well as drug resistance, and hybrid multidisciplinary treatments.

## 3. Glioma-on-Chip Platforms

### 3.1. Therapeutic Applications on Glioma-on-Chip Platforms

**Figure 1:**
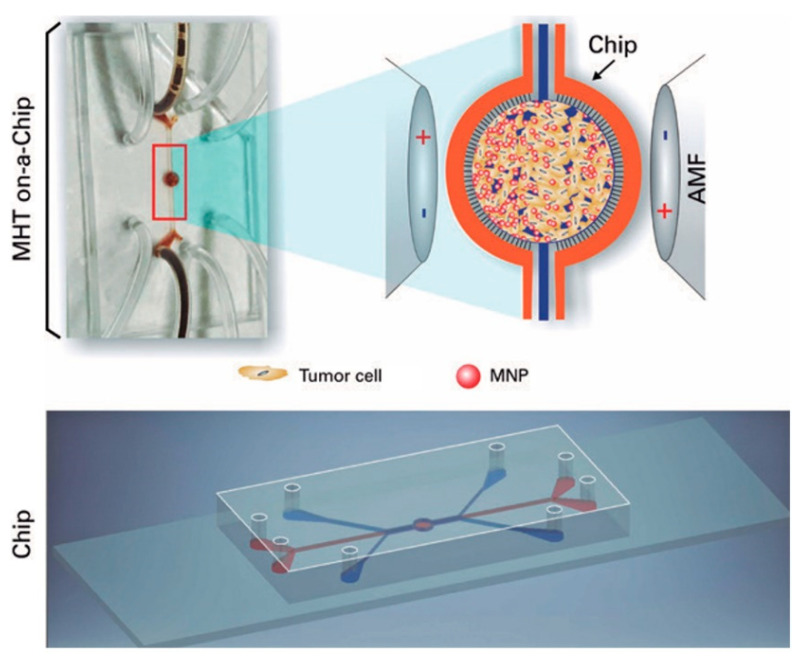
On-a-chip magnetic hyperthermia therapy for GBM. Magnetic nanoparticles interacted with GBM cells in the central region of the chip design, while being exposed to the magnetic field that caused an increase in the heat directly affecting the tumor cells by magnetic nanoparticles [34]. Reproduced with permission from [34].

Potential therapies of gliomas could be evaluated and optimized by utilizing specific tumor-on-a-chip models, minimizing the utilization of in vivo animal models. In a study by Mamani et al., the magnetic hyperthermia therapy (MHT), that induces heat for treatment of cancer cells by magnetic nanoparticles (MNP), was implemented using a microfluidic device with culturing the GBM tumors on a chip platform. With the use of MHT, tumor cells interacting with MNP were exposed to the alternating magnetic field and the viability of those cells was investigated between 41 °C to 43 °C. 2D culture is not an efficient way to evaluate the cellular response to the therapies due to the failure of representing the actual in vivo structure and dynamic condition. In this study, a microfluidic system was proposed, including two compartments separated by a porous interface, allowing cell-cell interactions and 3D cell culture which is unproducible in 2D cultures. Moreover, microchannels enabled fluid flow throughout the culture medium, mimicking the dynamic tumor environment in vivo by managing the flow of magnetic nanoparticles targeting cancerous cells. However, the drug screening results can deviate from in vivo results in 2D cultures owing to the lack of efficient flow of drugs [34]. Unlike 2D cell cultures, cell cultures that grew in an appropriate cavity in the chip showed the highest resemblance to the in vivo tumor cells. Aminosilane-coated MNP, making MNP biocompatible, were used for treatment (Figure 1). The results demonstrated that, after 30 min of on-chip treatment, magnetic hyperthermia treatment was 100% effective in mass death of GBM cells (C6 rat glioma cells) [34].

**Figure 2:**
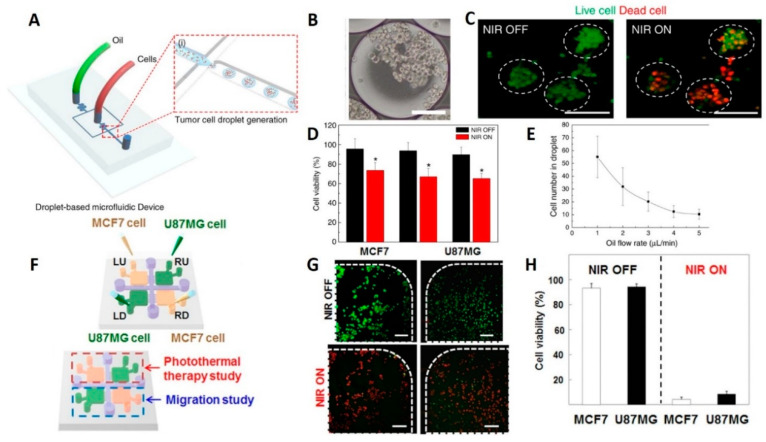
Glioma-on-chip platforms for photothermal therapy. (**A**) Schematic illustration of the generation of the 3D tumor spheroids. (**B**) Microscope image of the encapsulated cell in the generated droplets (scale bars are 100 μm). (**C**) Fluorescence microscopy images showing the effects of NIR laser treatment on brain tumor spheroids. Live cells were stained with calcein AM (green) while dead cells were stained with ethidium homodimer (red) (scale bars are 100 μm). (**D**) Results of NIR laser irradiation on the cell viability of U87MG brain tumors after being treated with rGO-BPEI-PEG nanocomposites (red bars) and control experiments without the addition of rGO-BPEI-PEG nanocomposites (black bars). (**E**) Correlation of oil flow rate in the junction to the concentration of encapsulated cells in a single droplet [35]. Reproduced with permission from [35]. (**F**) Schematic view of hydrogel microfluidic co-culture device. (**G**) Photothermal therapy of cancer cells. Fluorescent images of cell viability in square-shaped microchambers (green dots represents live and red dots are dead cells) (scale bars are 100 μm) (**H**) Quantitative results of the viability of U87MG and MCF7 cancer cells using CCK-8 assay [36]. Reproduced with permission from [36].

Lee et al. used a droplet-based microfluidic system to form 3D tumor spheroids (100–130 μm in diameter) including U87 GBM cells for photothermal therapy (Figure 2A–E) [35]. The size of spheroids could be controlled either by manipulating droplet volumes depending on the flow rate in the junction, or by altering the cell concentration. The microfluidic chip was able to create 42,000 droplets (~20% higher generation yield than similar setups), in 10 min, while 80% of droplets contained spheroids, when the oil flow rate is maximum (50 μL/min).

Drug studies were conducted with thapsigargin treatment for neural stem cell (NSC)-derived neurospheres made by this chip system to investigate the effects of the neurotoxin on neuronal cells. Using immunostaining, thapsigargin-induced neurite damage was assessed, revealing that the neurite length was reduced from 42 to 14 μm after treatment. Therefore, the droplet-based chip system is appropriate for neurological disease drug studies. Moreover, the viability of the tumor spheroids, after treatment by rGO-BPEI-PEG nanocomposites, declined from 91% to 55% following near-infrared (NIR) laser irradiation which proved that rGO-BPEI-PEG nanocomposites are a potential photothermal therapy (PTT) agent for 3D tumor spheroids acquired from droplets [35].

A GelMA hydrogel-based co-culture tumor-on-a-chip model was developed by Lee et al. to study cancer metastasis and evaluate the efficacy of PTT on GBM (U87MG) and breast (MCF7) cancer cells (Figure 2F–H) [36]. Both PTT and migration studies were conducted in a single microfluidic platform developed using two-step photolithography. After NIR laser irradiation, cell viability of MCF7 and U87MG cells, that were treated with 20 *v*/*v*% gold nanorods, was sharply decreased from ~90% to <10%, regardless of cancer cell types. Conversely, it was demonstrated that independent treatment of cancer cells with either gold nanorods or NIR laser irradiation resulted in poor efficiency with high cancer cell viability after treatment. Besides, no cytotoxic was reported for the NIR irradiation process and gold-nanorods [36].

### 3.2. Glioma-on-Chips for Reconstituting Glioma Microenvironment and Studying Cell Behaviors

**Figure 3:**
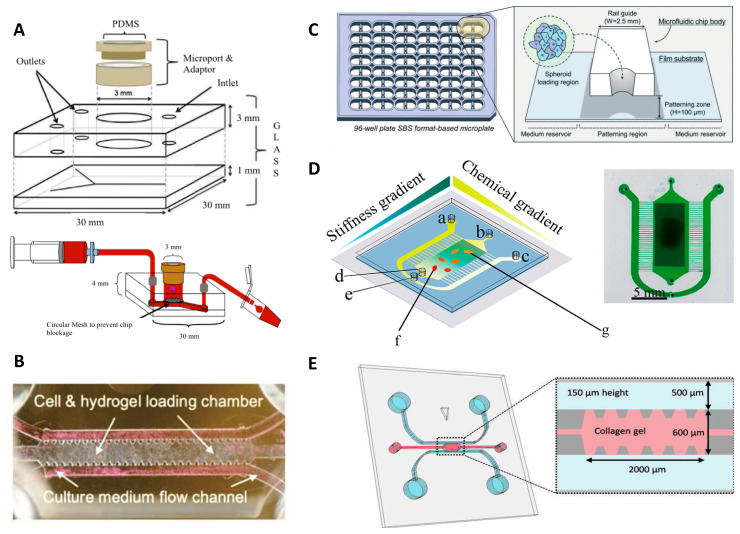
Reconstituting glioma microenvironment and studying cell behaviors via on-chip platforms. (**A**) Representative images of the microfluidic culture system setup [37]. Reproduced with permission from [37]. (**B**) GBM-microvasculature-on-a-chip model. The microfluidic system contained three channels; one for cell embedded hydrogels and the other two channels for culture medium flow [38]. Reproduced with permission from [38]. (**C**) Sphero-IMPACT 96-well microfluidic system. The area for cell culture was located between the medium reservoirs [39]. Reproduced with permission from [39]. (**D**) Conceptual image of microfluidic chip platform allowing stiffness and chemical gradients simultaneously. a, b and c illustrate inlets; d and e show outlets of cell culture and solutions, respectively; f and g represent culture area that includes U87 cells loaded with EGF [40]. Reproduced with permission from [40]. (**E**) Microfluidic chip for cell invasion study. Cells were seeded into the two parallel channels demonstrated with blue, and pink area that represents the area containing type I collagen [41]. Reproduced with permission from [41].

Olubajo et al. took 128 biopsy samples from the cancer patients and successfully cultured them for virtually 3 days in the ex-vivo fluid flow environment, which provides continuous nutrient circulation and waste removal. The microfluidic culture system, including inlet and outlet channels, was fabricated using standard photolithography and wet etching techniques. The proposed chip was created by combining two different layers of glass to connect the microchannels and the tissue chamber (Figure 3A). Flow cytometry analysis indicated a 61.1% cell viability rate, after 72 h, for specimens cultured on the microfluidic platform, compared to that of fresh initial tissue (68.9%), pointing out a reasonable approximation of obtained data from glioma-on-chip platform to the in vivo GBM tissue for mimicking biology and natural structure of the tumor tissue [37].

GBM tumors can spread to the entire brain parenchyma hijacking the existing tracks in the brain, such as the perivascular niche (PVN) or myelinated tracts. A GBM-microvasculature-on-a-chip platform, as PVN model, was developed by Xiao et al. that mimicked the in vivo characteristics of GBM and enabled the evaluation of ex vivo dynamics of the tumor cells as well as the function of brain tumor stem-like cells (BTSC). The chip model included three distinct channels; one of them offered fibrin gel loading containing cells, while the other two channels served as medium flow channels (Figure 3B). Using this platform, the cell migration direction and cell adhesion were correlated to the connection between collagen IV and tumor cells on the vessel surface. Furthermore, the 3D engineered platform indicated that the co-localization of the tumor in the perivascular region was related to the expression of platelet-derived growth factor receptor alpha (PDGFRA). The developed model offered an opportunity for patient-specific tumor investigation and observation of tumor cell heterogeneity, as an appropriate platform in the ex vivo experiments [38].

While co-option of blood vessels is one important factor during tumor invasion, tumors also recruit new blood vessels causing angiogenesis; another challenging biological mechanism for cancer therapy and drug screening. Ko et al. developed a 96-well plate 3D tumor-spheroid-on-a-chip cell culture platform (U87MG), called Sphero-IMPACT, using polystyrene material instead of PDMS due to limitations associated with manufacturing (Figure 3C) [39]. The platform was equipped with a perfusable network of microblood vessels to promote the versatility of the model. Spheroid-based models recapitulate biological behaviors such as invasion, migration, and cancer vascularization, enabling preclinical pharmacological drug studies. The images of the co-culture platform including 500–600 μm-sized tumor spheroids, formed by U87 cells, were taken for 4 days. The platform was able to screen effects of TNF-α and TGF-β1 on cancer metastasis, displaying an improvement of the invasion and migratory potential of U87 cancer cells 48 h after applying TNF-α and TGF-β1. Besides, the outcomes of angiogenesis assays and drug screening validation showed that anti-angiogenic drugs, such as bevacizumab and sunitinib, reduced the total vascular network area, sprouting length, and the number of sprouts in the co-culture environment [39]. In another study, Lee et al. developed an ECM-based hydrogel-incorporated microfluidic system to investigate the cellular behavior of the glioma cells. The cell migration was tracked under diverse conditions, such as with/without tissue inhibitor of metalloproteinases (TIMP) or with vascular endothelial growth factor (VEGF). The fluidic platform provided a high viability rate for gliomas (75–85%), generating a control biomimetic matrix for cancer cell studies [42]. To examine the cell behavior of glioma cells, a polyacrylamide hydrogel-based microfluidic chip was fabricated that gives the opportunity to analyze the cell migration of the U87 GBM cells by generating orthogonal chemical stimulation and controllable stiffness gradient (Figure 3D) [40]. Increasing hydrogel stiffness can regulate cells (e.g., morphology, migration, and differentiation). The moving mask-based photopolymerization technique was used to produce a linear and well-defined stiffness gradient [43,44]. By moving an opaque mask overlying the acrylamide/bis-acrylamide solution, the stiffness gradient profile on the hydrogel was acquired. A syringe pump was used for controllable moving speed which resulted in monotonically augmented stiffness with irradiation time. It was reported that hydrogel stiffness promotes chemotaxis of cells, whereas the Epidermal Growth Factor (EGF) gradient accelerates cell migration [40]. Another platform quantified the proliferation and migration potential of primary patient-driven GBM cells through immunostaining with Ki-67 and Hoechst. The assay was able to classify patients based on progression-free survival with 86% accuracy [9]. A collagen-based GBM-on-a-chip platform was utilized for mimicking the formation of pseudopalisades, a histologic hallmark of GBMs that appear under hypoxic intratumoral environment. The GBM chip model was tested in the hypoxia condition for U-251 MG GBM cells. This experiment indicated that lack of O_2_ and nutrients bring about a migration process, leading to pseudopalisade formation [45].

Chonan et al. by proposed a 3D microfluidic coculture model (Figure 3E) to mimic 3D brain tumor microenvironments by type I collagen and human umbilical vein endothelial cells (HUVECs). Collagen Type I was injected into the gel region and incubated (30 min) to form a hydrogel which was used as the scaffold material in the microfluidic chip. 1 × 10^6^ cells/mL of HUVEC cells was injected in one of the microchannels which was already filled with the endothelial growth medium (EGM2). After culturing for 3 days, 1 × 10^4^ cells/device glioma initiating cells (GICs) cells were added into the other channel. After attachment of GICs to the sidewall of the gel scaffold, the culture media in the channels for HUVECs and GICs were replaced with endothelial growth basal medium based media on a daily basis. Using this model, it was revealed that HUVECs enhanced the invasive property of the glioma cells. Furthermore, it was shown that genes related to invasiveness were upregulated with HUVEC cells in their microenvironment [41].

### 3.3. Glioma-on-Chips for Drug Studies

Lin et al. elucidated that endothelial cells in the perivascular microenvironment promote resistance to drug response of glioma stem cells (GSC), which was challenging to show in conventional 2D culture environments that do not recapitulate interactions of endothelial cells and glioma cells [46]. Accordingly, GSCs could be treated in the nature-mimicking glioma PVN on the microdevice while maintaining cancer stem cells at the multipotent status owing to cell-cell communications. The microchip enabled the study of the neurosphere form of GSC, clarifying the crucial role of endothelial cells in drug resistance. Investigating the effect of TMZ on GSCs in the biomimetic systems, it was reported that treatment with 800 μM of TMZ resulted in a 50% higher death rate of GSCs. After 72 h of coculture of the endothelial cells and GSCs, the cell survival rate as well as expressions of CD133 and Nestin on GSCs-only culture was weaker than cocultured GSCs. In the case of treatment of the GSCs by TMZ, 7-methylguanine (7-MeG) and 6-O-methylguanine (6-O-MeG) are the two biomarkers of chemoresistance diagnosis [22]. Using the proposed OOC platform, the expression levels of 7-MeG and 6-O-MeG culture were observed to be higher than cocultured GSCs, highlighting the role of endothelial cells in chemoresistance and glioma therapies [46].

**Figure 4:**
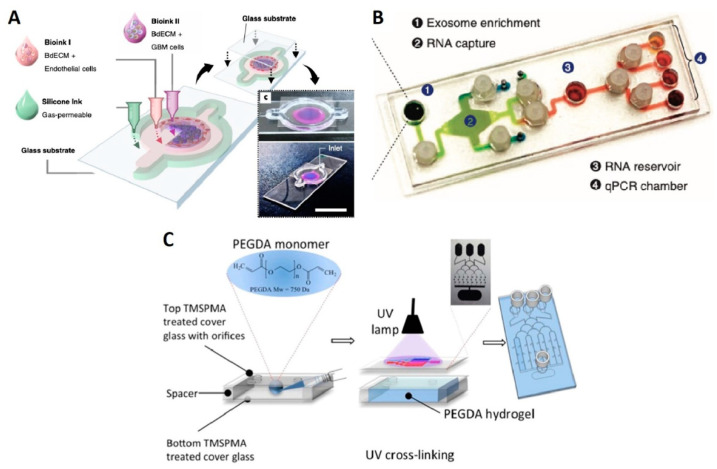
Glioma-on-chips for drug studies. (**A**) 3D bioprinted GBM-on-a-chip between the glass substrates (slides). Bioink I containing BdECM and vascular endothelial cells; Bioink II containing BdECM and GBM cells; and gas permeable silicon ink constructing the ex vivo microfluidic platform [47]. Reproduced with permission from [47]. (**B**) Image of exosomal RNA assay platform. The platform included four different regions for four distinct functions to analyze the cancer exosome [48]. Reproduced with permission from [48]. (**C**) Representative illustration of the fabrication of PEGDA hydrogel-based microchip for drug screening [51]. Reproduced with permission from [51].

Ex vivo GBM-on-a-chip models allow the development of more convenient personalized cancer therapies and identification of patient-specific drug sensitivity. A platform developed by Yi et al. representing patient-specific resistances to therapies and can be used to determine drug combinations for the more effective tumor-killing performance of treatment [47]. As bioinks, the GBM and HUVEC cells were used with silicon ink and brain-derived extracellular matrix (BdECM) for printing ex vivo platforms on the glass substrate (Figure 4A). The combination of the chemotherapy and chemoradiotherapy drug candidates were examined on the tumor cells through the cancer-mimicking chip platform, facilitating patient-specific prediction of the treatment outcome. While both hydrogels used in this experiment had > 90% cell viability, after 10 days, the cell proliferation rate in BdECM gel was higher than that in collagen gel. Additionally, 3 days after culturing, lower expression levels of genes, that encode the ECM-remodeling proteins (MMP9, MMP2, FN, MMP1, and PTK2) and pro-angiogenic factors (IL8 and VEGFA) were reported for collagen gel compared to BdECM gel. Besides, compared to BdECM gel, CD31^+^-endothelial cells produced less active tubule networks in the collagen gel, after 14 days, while BdECM gel also displayed a superior capacity of the angiogenesis of HUVECs. Chemoradiation (CCRT) was applied using TMZ on the chips that were made from patient-derived GBM cells. Following drug treatment, the chips made of patient cells with the highest survival rate yielded < 40% cancer cell viability in response to the drug therapy, whereas chips with patient cells with the lowest survival rate showed > 60% cancer cell viability, pointing out the ability of this platform to predict malignancy level [47].

Exosomes play a central role in the glioma tumor microenvironment. For instance, exosomes can contribute to cell-to-cell communication by delivering bioactive molecules (e.g., nucleic acids, lipids, and proteins), or can serve as a cancer biomarker. Moreover, studies showed that exosomal non-coding RNAs or microRNAs (e.g., miR-1, miR-21, miR-221, lnc-RNA-AHIF) regulate the cellular behavior of the glioma such as angiogenesis, proliferation, invasion, immunosuppressive behavior, and resistance to chemotherapies, highlighting the importance of exosomal studies to promote glioma treatments. Exosomal ribonucleic acid (RNA) analysis of such enzymes was conducted by Shao et al. on a microfluidic chip (Figure 4B) with 100 μL of serum, in 2 h. This platform was able to separate exosomes with 93% accuracy and extract RNA 50% better than commercial kits [48]. Uncontrolled diffusion of molecules between channels can bring about contamination and false results especially in hydrogel matrix based microfluidic chips. A tumor-on-chip platform, with an ability to avoid cross-channel drug diffusion, was produced to evaluate the drug response of tumor cells prior to clinical treatments. Using this platform, GBM cells can be isolated from the patients and cultured in the spheroid forms, while the different concentrations of the drugs, generated by microfluidic channels, can be tested on them. Chemotherapy agents such as TMZ and bevacizumab were studied, reporting that primary GBM tumors showed more efficient drug response to the combined form of the drug rather than single TMZ [49].

Another microfluidic GBM model was applied to examine the drug-related autophagy mechanism. In reference to the results, TMZ and simvastatin, which trigger the apoptosis, were not only less effective on the cells in the 3D microfluidic system, but also the rate of cells leading to apoptosis by these drugs was quite low in 3D. The tumor-on-a-chip model was a PDMS-based microfluidic chip with hydrogel-based channels comprised of GBM cells and stroma. This system was also a practical alternative for comparing the results of diverse behavior of the cells in 2D and 3D platforms for various ex vivo drug treatment studies [50]. As another high-throughput drug combination study by microfluidic systems, poly(ethylene) glycol diacrylate (PEGDA)-based 3D tumor-on-chip model was used to culture U87 human GBM cells to test pitavastatin and irinotecan. The main advantages of this platform were offering a test of several drug combinations at the same time and being convenient for spheroid formation. The fabrication of the chip platform was based on UV-lithography of PEGDA-hydrogel, and the preparation of this platform did not need silicon wafers and molding step, unlike PDMS-based chips (Figure 4C) [51]. Besides, enhancing immunotherapy strategies for genetically nonhomogeneous tumor environments is challenging. GBM-on-a-chip developed by Cui et al. were utilized to provide an insight into the tumor and immune interaction by primary GBM tumors from patients in real-time [52]. In order to screen immunostained GBM, T-cells, and tumor-associated macrophages (TAM cells), the inverted fluorescent microscope was used. In addition, quantification of cell migration behavior of CellTracker Green-labeled allogeneic CD8+ T-cells was performed by acquiring time-lapsed image stacks every minute, for 2 h, and at three different positions in each microfluidic chip. To calculate the cell migration speed and linked up to represent the migration trajectories, ImageJ (NIH, Bethesda, MD, USA) was employed to track the cell centroids at various time points of the same cell. Immunosuppressive tumor niche demonstrated that targeting of both programmed cell death protein-1 and CSF-1R signaling contributed to a more effective GBM treatment [52]. Table 1 presents the requirements of glioma-on-chip platforms and how microfluidic chips can contribute to the field.

## 4. Cell Sources for Organ-on-Chip Platforms

The source of cells used in culture is decisive for the relevance and the predictive value of OOC models. Stem cells, primary cells, and cell lines are the most suitable candidates for OOC applications. Stem cells last longer in cell cultures, can differentiate into many cell types, recreate various organ-like structures with the same genetic background, and are readily available [59,60,61]. However, current technological limitations have restricted the application of stem cells for OOC platforms. The majority of current OOC platforms use primary cells and/or cell lines.

### 4.1. Primary Cells

Primary cells can be obtained by removal of the biopsy/pieces from organs or tissues, in aseptic conditions, and then acquiring cells using enzymatic, chemical, or mechanic digestion approaches [62]. Neurovascular units, liver, kidney proximal tubule, lung, and heart valves are a number of organs cultured on chips using primary cells [63,64,65,66,67]. Nonetheless, due to the difficulty of obtaining human primary tissue, a number of these studies used co-cultures with established cell lines (required to model cell- or organ-level interactions more accurately) or animal primary cells [63,64,65]. The main analogy between primary cells and tissue biopsies is being derived directly from adult tissue, potentially providing more precise information on the biological characteristics of mature tissue [59]. Tissue biopsies have additional advantages of partially possessing 3D tissue structures (i.e., natural organ-specific extracellular matrices) which 2D cultures miss [68]. Inconsistent quality and surviving for limited time ex vivo (~48 h) are major challenges of primary cells for OOC applications [68].

### 4.2. Cell Lines

The term cell line refers to a defined population of cells that can maintain the stability of certain functions and phenotypes under in vitro culture conditions for an extended period of time [69]. Cell lines were utilized in OOC platforms including liver, placenta [70,71], and multi-organ chip systems, such as combinations of the adipose tissue, bone marrow, skin, kidneys, gastrointestinal tract, liver, and lung [72,73]. Primary cells are similar to cell lines in lacking the natural extracellular matrix and being a source of more mature cells [68]. However, although, compared to primary cells, cell lines produce reproducible results as a consequence of having a more homogenous population, unlike stem cells, tissue biopsies, and primary cells, cell lines lack the patient-specificity, limiting their applicability for disease modeling [68,74]. Besides, cell lines have limited ability for toxicity tests owing to the induction of overexpression of proteins involved in specific toxicity-related pathways [74]. In a study conducted to compare the expression of proteins in primary hepatocytes and hepatoma cell lines, downregulation of drug-metabolizing enzymes and normal metabolic pathways to favor cell-cycle-associated proteins were observed in cell lines, highlighting inaccuracy of cell lines for tissue function recapitulation [75]. Hence, despite being widely in use for OOC applications, cell lines face challenges in mimicking natural physiology.

## 5. Microfluidic Chip Fabrication

Owing to high analytical potential and the increasing presence of microfluidic devices in scientific studies [76,77,78,79,80,81], a diversity of fabrication methods are proposed for microchip production [82,83,84,85,86]. For 3D laminate microfluidic devices are produced by stacking (using adhesives or thermal bonding) independent 2D cut layers (e.g., interface, flow, and bottom layers) to form a final 3D structure [87]. This is a rapid, inexpensive, and simple method that needs precise alignment of different layers with size features ranging from 50 to 200 μm, mostly limited by the resolution of available cutting methods [82]. Laser cut is a promising solution to produce smaller size features (as small as 25 μm) in laminate microfluidic devices [88]. The working principle of replica molding (i.e., soft lithography [89]) is based on consecutive steps of photolithography, photoresist curation (e.g., SU-8), and PDMS pouring to form desired geometries [90,91,92]. Despite being able to produce submillimeter features (30 nm to 100 μm), mold fabrication requires cleanrooms for the fabrication process, increasing the cost and complexity of production. Thenceforth, the produced mold can be replicated, without cleanroom, in larger quantities with a simple and low-cost soft lithography process [93,94]. Injection molding (known as microinjecting) uses thermoplastics to produce precise dimensions (in the order of 25 μm) with high throughput [82,95]. However, limited material choice (thermoplastics), inability to produce designs with undercut features, and high cost of molds are challenges associated with injection molding [96,97].

3D printing, a subset of additive manufacturing also known as rapid prototyping, is an emerging technology that translates computer-aided designs to 3D structures in a layer-by-layer fashion [82,98,99,100]. Since computer designs can be produced directly in this method, high expertise in micromanufacturing is not needed, enabling low-skill researchers to perform intricate design iterations/modifications with no need to third-party manufacturing companies [101,102]. Commonly used 3D printing techniques for microfluidic chip fabrication are extrusion-based methods (e.g., fused deposition molding (FDM, 50–200 μm resolution)), light-induced methods (e.g., stereolithography (SLA, 10 μm resolution), two-photon polymerization (MPP/TPP, 100 nm to 10 μm resolution), digital light processing (DLP, 25–100 μm resolution), polyjet methods (25 μm resolution), and powder-bed based methods with 50–250 μm resolution (selective laser sintering (SLS), and selective laser melting (SLM)) [103,104,105]. Table 2 summarizes the important features of 3D printing methods.

## 6. Conclusions and Future Perspectives

Gliomas are tumors originated from the glial cells, such as astrocytes, oligodendrocytes, and ependymal cells within the central nervous system which accounts for 80% of all malignant brain tumors. Microfluidic OOC technologies have great potential for tumor studies to illuminate the tumor biology, underlying reasons of gliomagenesis, and offer an effective in vivo-like platform to find optimum multimodality therapies (e.g., radiotherapy, drug treatments) [108]. Although there are limitations related to the nonhomogeneous tumor environment, it has been demonstrated that OOC platforms can be used for probing potential therapies such as MHT [34], reconstituting tumor microenvironment including peripheral tissues and cells [48,109], and studying cell migration [9], invasion [41], angiogenesis [39], autophagy mechanisms [50] and drug responses [47,48,49]. Furthermore, glioma OOC platforms ensure decreasing of the usage of in vivo animal models [34], and facilitate medical decisions to find the most effective drug combinations [47]. 3D microfluidic platforms with continuous perfusion provide a co-culture with high cell viability, giving an opportunity to examine the correlation between the distinct cell types in biological mechanisms such as cell invasion [41], angiogenesis that are challenging during the chemotherapy [39]. Moreover, recent progress in culturing patient-driven stem cells on OOC platforms is promising regarding the development of personalized cancer therapies and patient-specific drugs.

Providing uniform test standards and stable readouts should be addressed in the future to enable reliable results and enabling OOC chips to get approval from the U.S. Food and Drug Administration (FDA). In order to obtain high throughput, stable readouts of various physiological processes that are taking place on a chip, molecular reporters and nanoscale biosensors can be utilized [110,111]. Also, contemporary microscopes are able to record data, either images or videos, from chip platforms at a high pace. However, classification, quantification, and interpretation of acquired data by clinicians are error-prone, labor-intensive, and time-consuming, hampering timely diagnosis and decision making. Hence, machine learning (ML) techniques can be integrated with glioma-on-chip platforms for accurate, rapid, and autonomous analysis of available data without being explicitly programmed by humans [112]. Although the immune system of the human body plays a key role in cancer, OOC systems which are taking into account immune cells are rare [10,110], highlighting the importance of developing glioma-immuno-on-chip platforms. A noteworthy limitation of OOC platforms is the minute volume of secreted cellular products due to the small number of cultured cells on a chip, complicating detection and sampling of desired bioparticles [56]. Possible solutions can be decreasing the flow rate of perfusion, increasing the length of the culture channel, and having multiple culture chambers in parallel. One of the critical advantages of OOC cell cultures is the continuous perfusion through microchannels which can promote cell proliferation and viability. While perfusing multiple channels simultaneously, active perfusion with micropumps brings about additional cost and complexity to on-chip platforms. Thus, gravity-driven, passive perfusion approaches, based on differential fluid pressure in the reservoirs, can provide a simple, low-cost still high-performance platform without the need for proficient users to conduct the experiment [10].

## Figures and Tables

**Table 1:** Requirements of glioma-on-chip platforms and the possible contribution of microfluidic chips [53,54,55,56,57,58].

Requirement	The Advantage Offered by Microfluidics	End-Application
Nutrient supply and waste removal [54]	Perfusion-based culture system [53,54] Micropumps	Cell-cell interaction [53,54] Maintaining tissue structure and function [53] Drug tests [54]
CO_2_, O_2_, or N_2_ exchange [53,54]	PDMS-based microfluidic chips with gas permeability [53,54]	Higher cell viability and proliferation rate [54] pH regulation [53]
Visual analysis	Transparency [56]	Microscopic imaging
Dynamic condition	Microchambers with porous membranes [54]	Cell-cell interaction through the diffusion of small molecules Creating oxygen gradients and hypoxic conditions [54,55] Simulating cyclic strains that cell experience in vivo (e.g., during a heartbeat) [54]
ECM Matrix	Microchambers that can harbor ECMs (e.g., 3D bioprinted ECMs) [54]	Recapitulating tumor non-cellular environment [54]
Real-time analysis	Integration of biosensors to microfluidic chips [53,55]	Detection of cell adhesion, separation, and migration Eliminating the need for time-consuming, large cell population-based experiments [53]
In vivo-like condition	3D customizable structures Perfusion through microchannels Gas permeability of PDMS Deformable microchannels [54]	Simulation of responses of immuno cells to inflammatory stimulation under flow condition [57] Absorption of nutrients [54] Mimicking physiological architecture [53,54]
Single-cell analysis [53]	Culture of low number of cells in microchannels [53,58]	Study of cell behavior at the single-cell level, providing superior experimental resolution over macroscopic cell migration assays, such as the wound-healing assay [53]
Direct coupling to the downstream analysis system	Parallelization of various microfluidic chips [53] Integration with measurement equipment [53]	Rapid point-of-care analysis [54]
Co-culture with diverse cell types [55]	Cell culture in adjacent microchannels [53]	Study of interactions of different cell types More accurate drug screening High throughput experiments [55]
Reduced reagent/sample consumption [53,54]	Microscale channels and chambers [53]	Reducing the cost per test [53,54]
Contamination-free microenvironment [53]	Enclosed microchannels	High precision experiments
Compartmentalization through the diffusion of signaling molecules	Connected microchannel [54]	Resembling the physiological environment and cell-cell interactions [53,54]

**Table 2:** Commonly used 3D printing methods for microfluidic chip fabrication and 3D bioprinting.

Method	Resolution	Advantages	Drawbacks
FDM	50–200 μm [106]	Low cost—high speed—simplicity [106]	Limited mechanical properties and material (thermoplastics)—layer by layer finish [106]—An extrusion base process with high temperature (posing challenges for cells)—Rough surface [107]
SLA	10 μm [106]	Fine spatial resolution—high quality [106]—good surface quality—good precision [107]	Supports limited materials—slow printing—expensive [106]—poor biocompatibility—limited mechanical properties [107]
TPP/MPP [107]	100 nm–5 μm	High spatial resolution	Low build speed—limited material support
DLP [107]	25–100 μm	High printing accuracy—low cost—shorter build time than SLA—less affected by oxygen inhibition compared to SLA—better surface quality—low initial vat volume is needed	Limited mechanical properties
PolyJet [107]	25 μm	Fast—allow multimaterial printing	Low viscosity ink is needed
Powder-bed Based Methods (SLS-SLM) [107]	50–250 μm	Fine resolution—high quality—durable—large surface area, good for scaffolds of tissue engineering—good mechanical properties—less anisotropy	Slow printing—expensive—porosity—low mechanical properties—high power supply—high printing temperature—rough surface—poor reusability of unsintered powder
